# An Unusual Mechanism of Stroke in Neurobrucellosis: Middle Cerebral Artery Infarction Secondary to Direct Abscess Compression – A Case Report and Literature Review

**DOI:** 10.1159/crn/abyag015

**Published:** 2026-10-05

**Authors:** Ruba Adel Aweer, Majd A. AbuAlrob, Khaled Zammar, Ahmad Elsotouhy

**Affiliations:** a College of Medicine, QU Health, Qatar University, Doha, Qatar; b Department of Neurology, Hamad Medical Corporation, Doha, Qatar; c Department of Neuroradiology, Hamad Medical Corporation, Doha, Qatar

**Keywords:** Brain abscess, Stroke, Neurobrucellosis, Case report

## Abstract

**Introduction:**

Brucellosis is a zoonotic infection that remains endemic across the Middle East, Central Asia, the Mediterranean basin, and parts of Africa and Latin America. Neurobrucellosis (NB) develops in fewer than 5% of cases and constitutes the most severe systemic complication. Cerebrovascular involvement is uncommon and most often attributed to small-vessel vasculitis, immune-mediated endarteritis, or septic embolization. Ischemic stroke caused by direct mechanical compression of an intracranial artery by a Brucella abscess has not been previously reported.

**Case Presentation:**

A 47-year-old previously healthy male with occupational livestock exposure presented with sudden onset of left-sided weakness, facial droop, and global aphasia. Initial imaging demonstrated an acute right middle cerebral artery (MCA) territory infarct, and standard ischemic stroke management was initiated. Over the next 24 h, intermittent low-grade fever, persistent confusion, and focal motor seizures emerged despite levetiracetam. Subsequent magnetic resonance imaging showed a 2.5 cm rim-enhancing lesion in the right temporal lobe with central diffusion restriction, consistent with a pyogenic abscess. Magnetic resonance angiography demonstrated focal occlusion of the anterior segment of the right MCA, immediately adjacent to the abscess. Cerebrospinal fluid showed lymphocytic pleocytosis (36 cells/mm^3^, 85% lymphocytes) and elevated protein (112 mg/dL). Serum Brucella IgG returned positive on day 20, confirming neurobrucellosis. Triple antimicrobial therapy with intravenous ceftriaxone, oral doxycycline, and oral rifampicin produced gradual neurological improvement, and the patient was discharged on long-term oral therapy.

**Conclusion:**

This case illustrates a novel cerebrovascular mechanism in NB, in which mass effect from a peri-vascular abscess produced ischemic stroke. Coexisting perilesional inflammation likely contributed through a vasculitic component, and the two mechanisms are not mutually exclusive. The absence of systemic brucellosis features delayed recognition and underscores the protean clinical presentation of NB. Brucellosis warrants consideration in the differential diagnosis of acute ischemic stroke when imaging reveals concurrent intracranial infectious lesions, particularly in endemic regions. Early antimicrobial therapy is critical to limit progression and improve neurological outcomes.

## Introduction

Brucellosis is a zoonotic infection caused by gram-negative coccobacilli of the genus Brucella, with over 500,000 new cases reported annually [1]. Transmission to humans occurs through direct contact with infected livestock or consumption of unpasteurized dairy products [2]. The infection remains endemic across the Middle East, Central Asia, the Mediterranean basin, Latin America, and parts of Africa, where close human-animal interaction persists [3].

Clinical presentation is highly variable. Constitutional symptoms such as fever, malaise, arthralgia, and hepatosplenomegaly are common, although nearly every organ system can be involved [2, 3]. Neurobrucellosis (NB), defined as central nervous system involvement by Brucella species, develops in fewer than 5% of all cases [4]. It is regarded as the rarest yet most severe systemic complication.

NB presents with a wide neurological spectrum, including chronic meningoencephalitis, myelopathy, cranial neuropathies, psychiatric disturbances, and cerebrovascular events. Stroke attributable to NB is uncommon and most often attributed to small-vessel vasculitis, immune-mediated endarteritis, septic embolization from Brucella endocarditis, or, rarely, mycotic aneurysm rupture [5, 6]. Diagnostic delay is frequent because symptoms overlap with viral encephalitis and tuberculous meningitis.

This report describes a previously unreported mechanism of ischemic stroke in NB. A 47-year-old male presented with hyperacute global aphasia and left hemiparesis. Imaging revealed a right middle cerebral artery (MCA) territory infarct alongside a temporal lobe rim-enhancing lesion. Cerebrospinal fluid (CSF) analysis and positive Brucella IgG serology confirmed NB. The abscess directly compressed the segment of the MCA, producing the infarct. To the authors’ knowledge, no prior case has documented MCA territory infarction caused by mechanical compression from a Brucella-associated cerebral abscess. The case underscores the need for heightened suspicion of infectious etiologies in atypical stroke presentations.

## Case Presentation

### Patient Information and Clinical Findings

A 47-year-old previously healthy male with no known chronic illnesses presented to a primary healthcare center with acute slurred speech, left upper limb weakness, facial drooping, and confusion. The patient was afebrile and globally aphasic on arrival. Social history was initially limited by a language barrier. Subsequent collateral history from family members revealed occupational exposure to livestock at a rural farm setting (locally termed “Ezba”), with direct handling of animals.

Vital signs were notable for blood pressure 165/95 mm Hg, heart rate 88 beats per minute, temperature 36.9°C, and oxygen saturation 98% on room air. The patient was disoriented to time and place, globally aphasic, and unable to follow verbal commands. Meningeal signs were absent (no neck stiffness, no photophobia, negative Kernig, and Brudzinski signs). Systemic examination was unremarkable.

Neurological examination demonstrated left lower facial weakness, right homonymous hemianopia, and dysarthria. Power was reduced in the left upper limb (3/5) with preserved lower limb strength. Sensory function was intact, and the patient withdrew to noxious stimuli in all extremities.

### Diagnostic Assessment

#### Laboratory Studies

Routine hematological and biochemical investigations were within normal limits, with no leukocytosis or elevated inflammatory markers. Screening for human immunodeficiency virus, hepatitis B, hepatitis C, syphilis, and malaria returned negative.

A lumbar puncture was performed on day 3 of admission. CSF analysis revealed lymphocytic pleocytosis (36 cells/mm^3^, 85% lymphocytes), elevated protein (112 mg/dL), and normal glucose (68 mg/dL). Gram stain, bacterial culture, acid-fast bacilli smear, and fungal cultures were negative. CSF polymerase chain reaction panels for herpes simplex virus, varicella zoster virus, cytomegalovirus, Epstein-Barr virus, and enteroviruses were also negative. CSF flow cytometry showed no evidence of lymphoma.

Following clarification of livestock exposure, Brucella serology was requested. On day 20, serum Brucella IgG returned positive, confirming the diagnosis. Blood cultures remained negative throughout the admission.

#### Imaging

A non-contrast computed tomography (CT) scan of the brain on presentation showed subtle hypodensities in the right frontotemporoparietal region, suggestive of early ischemic changes within the right MCA territory. CT perfusion demonstrated a small infarct core surrounded by a larger penumbra. The patient was deemed ineligible for intravenous thrombolysis due to an uncertain time of symptom onset and established infarction on imaging.

Subsequent magnetic resonance imaging (MRI) of the brain provided definitive characterization (Fig. 1–4). Diffusion-weighted imaging and fluid-attenuated inversion recovery sequences confirmed an acute right MCA territory infarct (Fig. 1). A 2.5 cm rim-enhancing lesion was identified in the right temporal lobe, with multiloculated cystic components, surrounding vasogenic edema, and mild mass effect. Contrast-enhanced T1-weighted sequences demonstrated an irregular enhancing capsule with central diffusion restriction, consistent with a pyogenic abscess.

**Fig. 1.**
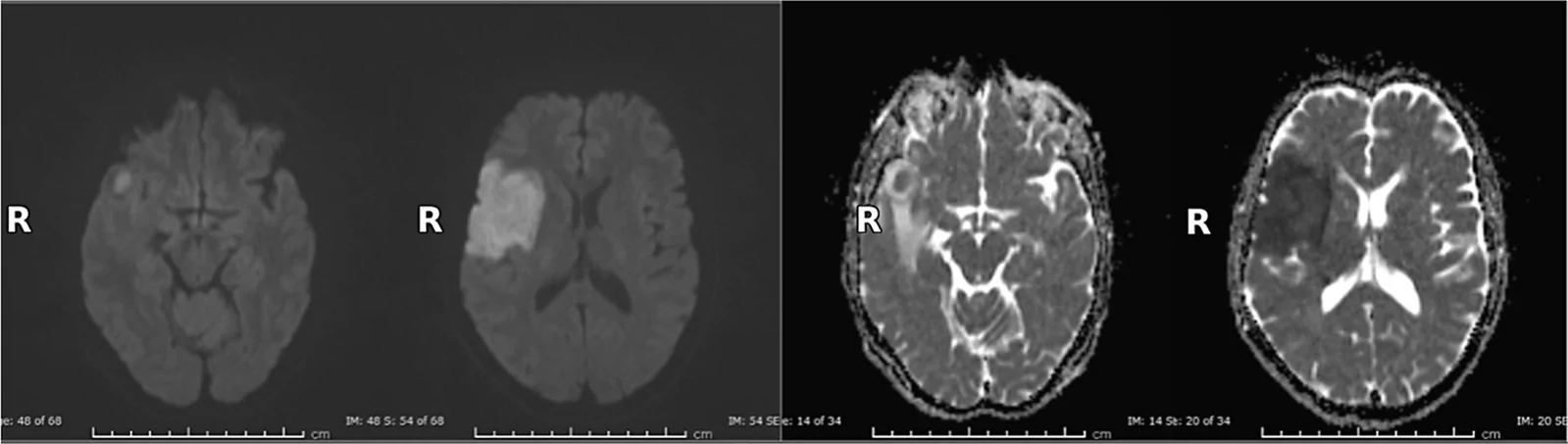
Axial diffusion-weighted imaging (DWI, left panels) and apparent diffusion coefficient (ADC, right panels) maps. Hyperintense signal on DWI in the right middle cerebral artery (MCA) territory with corresponding hypointensity on ADC confirms true diffusion restriction, consistent with acute ischemic infarction. Image orientation has been corrected; left and right hemispheres are now displayed in conventional radiological orientation.

**Fig. 2.**
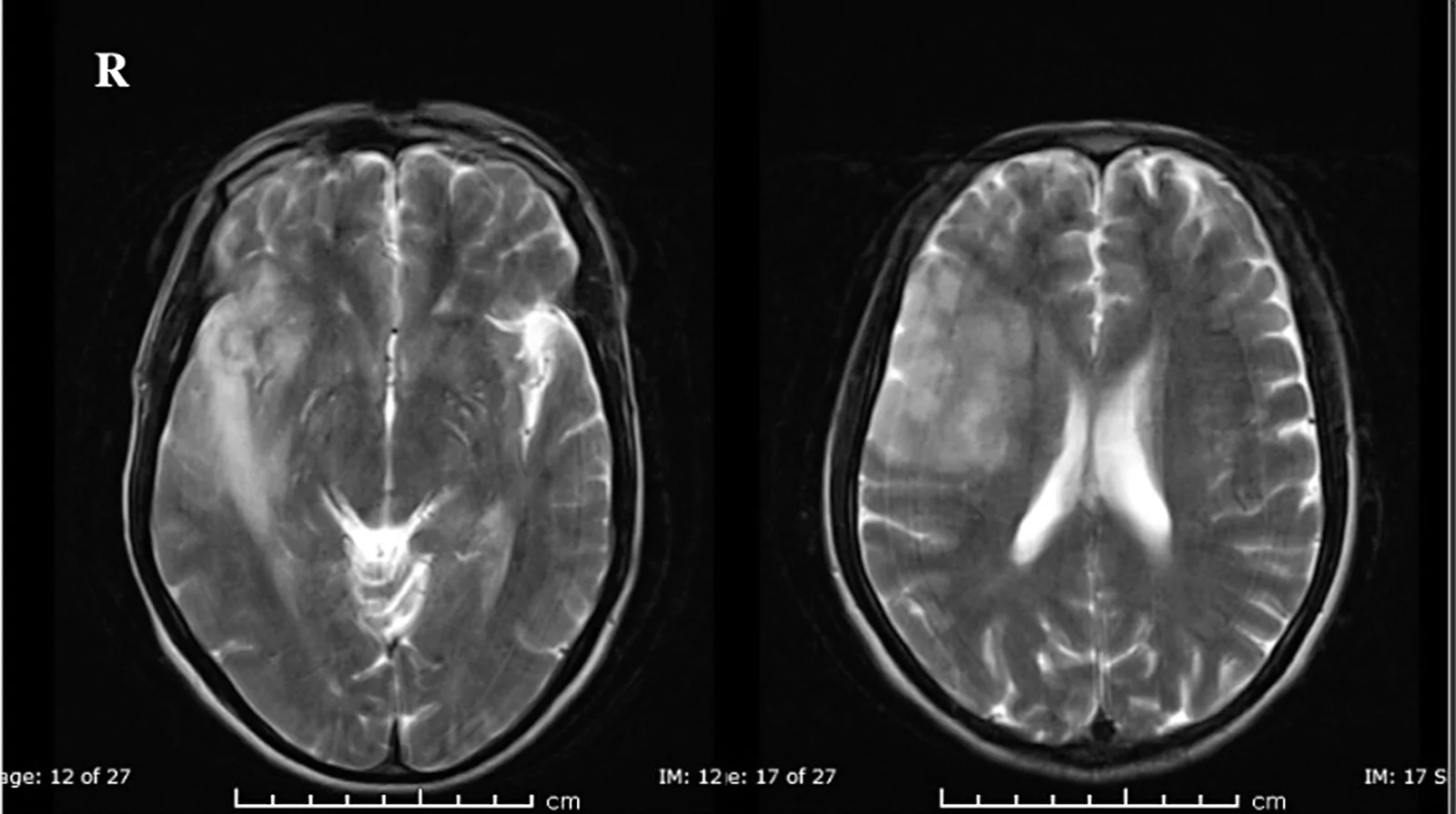
Axial T2-weighted MRI demonstrating a right temporal lobe rim-enhancing lesion with surrounding vasogenic edema and mass effect, consistent with a Brucella abscess. The lesion is hyperintense on T2 with marked perilesional edema causing partial compression of the adjacent lateral ventricle.

**Fig. 3.**
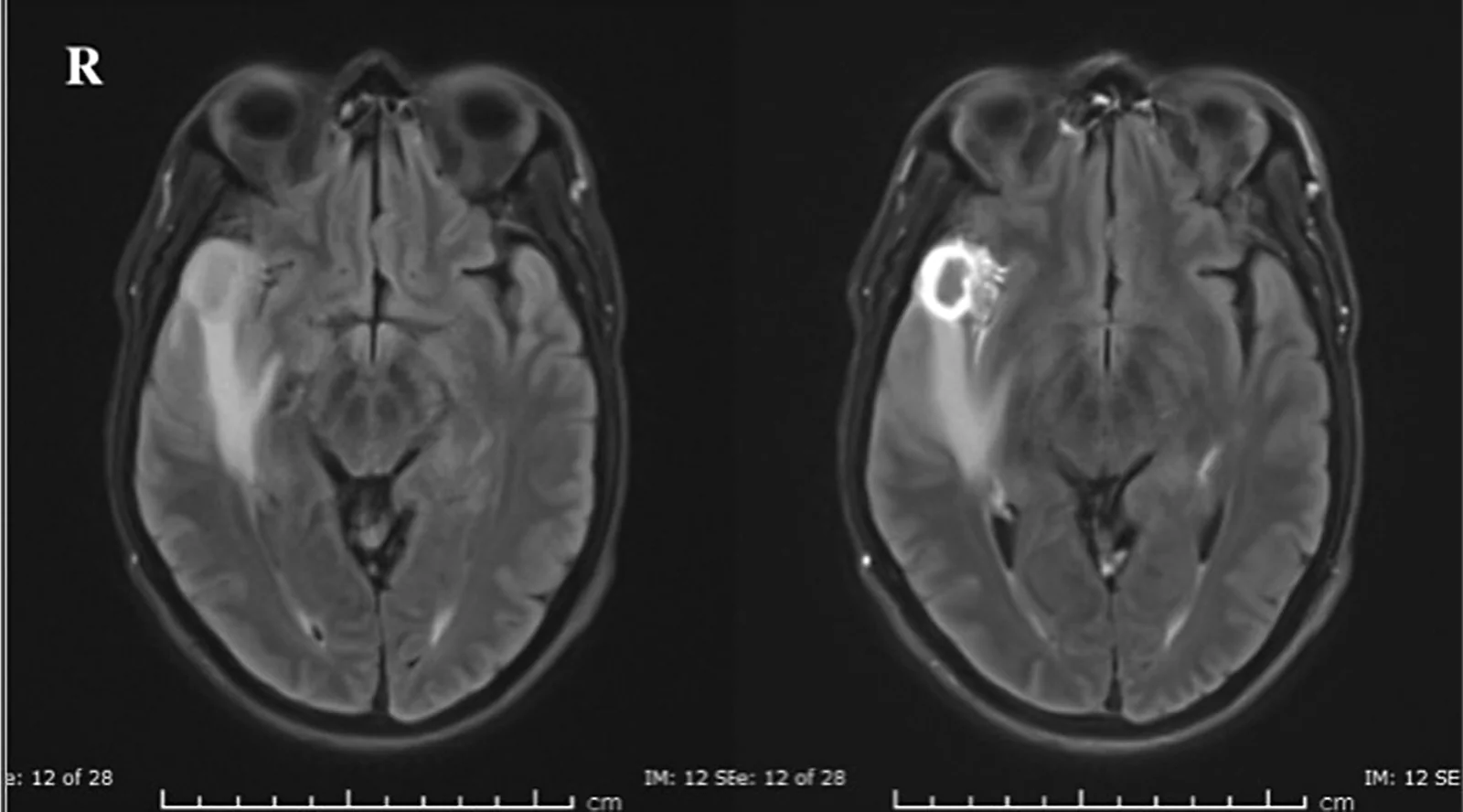
Axial fluid-attenuated inversion recovery (FLAIR) MRI showing the right temporal lobe ring-shaped hyperintense lesion with a central hypointense core and extensive surrounding vasogenic edema producing mild mass effect.

**Fig. 4.**
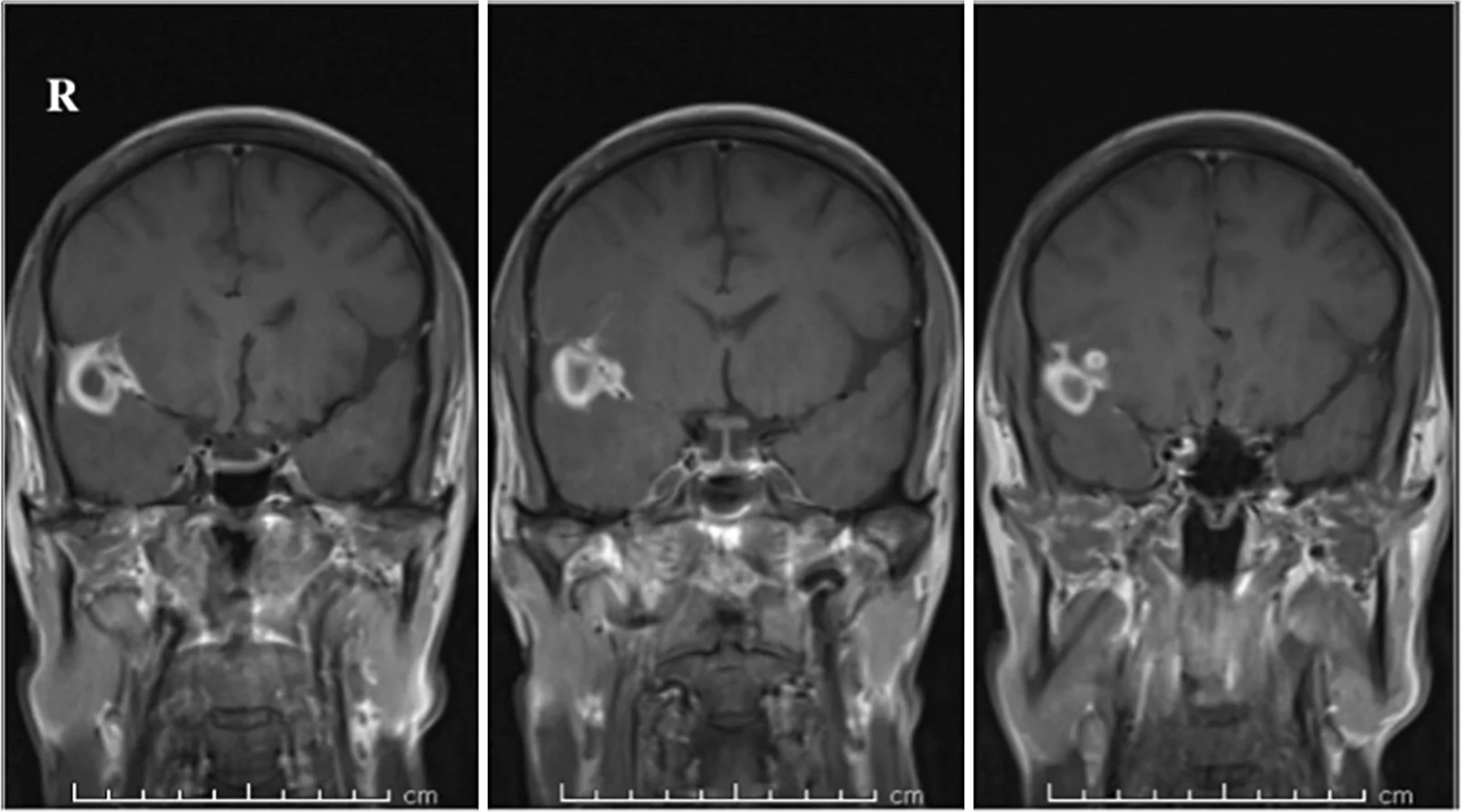
Coronal post-contrast T1-weighted MRI demonstrating a rim-enhancing lesion in the right temporal lobe with a central non-enhancing necrotic core and surrounding vasogenic edema. A well-defined enhancing capsule is evident.

Magnetic resonance angiography (MRA) demonstrated focal occlusion of the segment of the right MCA in close anatomic relation to the abscess (Fig. 5). Magnetic resonance venography excluded venous sinus thrombosis. No aneurysmal dilatation, vascular malformation, or large-vessel beading suggestive of widespread vasculitis was identified. Intracranial hemorrhage was absent.

**Fig. 5.**
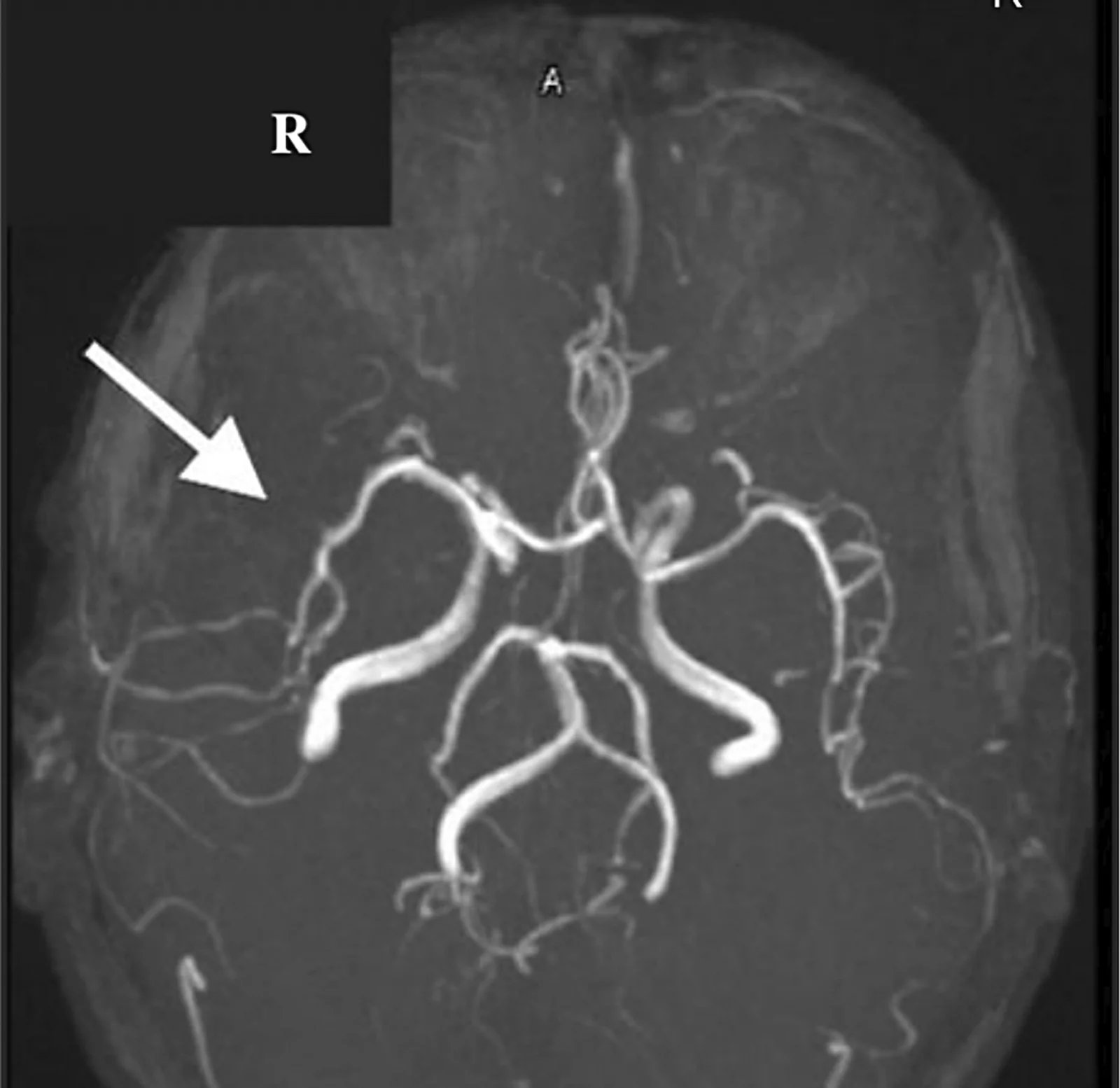
Magnetic resonance angiography (MRA) of the intracranial circulation. Focal occlusion of the anterior M2 segment of the right MCA is demonstrated (arrow), with preserved flow in the contralateral MCA and posterior circulation. The site of occlusion corresponds anatomically to the perilesional region of the right temporal lobe abscess shown in Fig. 2–4. No proximal large-vessel beading or aneurysmal dilatation is identified.

A whole-body positron emission tomography-CT demonstrated focal hypermetabolism at the site of the temporal abscess and identified no distant metastatic or infectious foci. Abdominal imaging incidentally revealed multiple hepatic cysts of indeterminate significance. Follow-up brain MRI at 3 weeks showed persistent abscess features with mild regression of perilesional edema and no new ischemic lesions.

### Therapeutic Interventions

The patient was admitted to the stroke unit. Acute management included permissive hypertension for 24 h, intravenous fluid resuscitation with normal saline, and close neurological monitoring. Following dysphagia screening, secondary prevention with aspirin 100 mg daily and atorvastatin 40 mg daily was initiated. Glycemic control was maintained, and early multidisciplinary mobilization with physiotherapy, occupational therapy, and speech and language therapy was commenced.

After identification of the temporal abscess in close proximity to the thrombosed M2 segment, neurosurgical consultation deemed surgical drainage unnecessary at that time. Empirical antimicrobial therapy was initiated with intravenous ceftriaxone (2 g twice daily), intravenous metronidazole, and adjunctive dexamethasone.

Following confirmation of NB, antimicrobial therapy was rationalized in consultation with infectious diseases. Metronidazole was discontinued, and oral doxycycline (100 mg twice daily) and oral rifampicin (600 mg daily) were added to ongoing intravenous ceftriaxone. The triple regimen continued through hospitalization, with planned long-term oral doxycycline and rifampicin for 6–12 months guided by follow-up imaging.

Supportive care included antihypertensive therapy with bisoprolol (2.5 mg daily), pharmacological prophylaxis of venous thromboembolism with enoxaparin, and short-acting agents (low-dose quetiapine, haloperidol, or lorazepam as required) for episodes of agitation. Recurrent focal motor seizures of the left upper limb were treated with levetiracetam, with adequate seizure control achieved during admission.

### Follow-Up and Outcomes

Serial MRI demonstrated progressive abscess regression without new ischemic foci. By discharge, the patient was alert, communicative, and ambulatory, with residual mild left facial weakness and mild dysarthria. He was discharged on long-term oral antimicrobial therapy with planned outpatient follow-up for clinical assessment and repeat imaging.

### Patient Perspective

The patient reported initial fear and confusion regarding the abrupt onset of weakness and aphasia, particularly given the absence of antecedent illness. He expressed gratitude for the multidisciplinary care provided and noted satisfaction with his functional recovery, while acknowledging persistent concern about residual speech difficulty. He emphasized the value of clear communication regarding the diagnosis and the rationale for prolonged oral antibiotic therapy.

The completed CARE checklist is available in the online “Supplementary material” (for all online suppl. material, see https://doi.org/10.1159/crn/abyag015).

## Discussion

This case describes a novel cerebrovascular complication of NB, in which an ischemic stroke arose from direct mechanical compression of the right MCA by an adjacent temporal lobe abscess. NB accounts for fewer than 5–10% of brucellosis cases and remains a diagnostic challenge given its protean presentation [4]. Cerebrovascular involvement within this subset is rare. Reported mechanisms include small-vessel vasculitis, immune-mediated endarteritis of perforating arteries, septic embolization in the context of Brucella endocarditis, mycotic aneurysm formation with subarachnoid hemorrhage, and cerebral venous sinus thrombosis (Table 1) [5–16]. Stroke arising from direct vascular compression by a Brucella abscess has not, to the authors’ knowledge, been previously documented.

**Table 1.** Reported cases of stroke and cerebrovascular involvement in NB

Author, year (Ref)	Country	Patient (age, sex)	Imaging findings	Proposed mechanism of stroke
Adaletli et al., 2006 [8]	Turkey	12 y, male	MRI: bilateral basal ganglia infarcts; vasculopathic arterial changes	Immune-mediated cerebral vasculitis
Gul et al., 2009 [17]	Multiregional	Pooled (*n* = 187)	Heterogeneous; stroke a minority	Meningovascular processes most often implicated
Ay et al., 2010 [5]	Turkey	24 y, female	MRI: multifocal infarcts; MRA: narrowing of MCA branches	Meningovascular inflammation
Ibrahimagić et al., 2017 [12]	Bosnia	Adult	Cerebral venous sinus thrombosis	CVST associated with NB
Turkoglu et al., 2018 [6]	Turkey	9 patients (mean 49 y)	Variable arterial vasculitis, granuloma, aneurysm	Small-vessel vasculitis (*n* = 5), large-vessel vasculitis (*n* = 3), pons abscess (*n* = 1)
Wen et al., 2021 [9]	China	38 y, male	Cerebral infarct with cord lesions	Meningovascular inflammation consistent with vasculitis
Patel et al., 2021 [15]	India	Adult	Acute ischemic stroke as presenting feature	Meningovascular inflammation
Guan et al., 2021 [16]	China	Adult	Subarachnoid hemorrhage; cerebral aneurysm	Mycotic aneurysm rupture
Alattas et al., 2022 [13]	Saudi Arabia	Adult	MRV: cerebral venous sinus thrombosis	CVST with rapid response to therapy
Liu and Gu, 2023 [7]	China	38 y, male	MRI: acute right MCA infarct; MRA: vessel narrowing	Small-vessel vasculitis
Bhat et al., 2024 [11]	India	Multicase	Ischemic infarcts with infective endocarditis	Septic emboli (valvular infection)
Fadul et al., 2024 [10]	Qatar	34 y, male	Embolic-pattern infarcts; cardiac vegetations	Septic embolism from Brucella endocarditis
Alhusseini et al., 2024 [14]	Syria	Young adult	Venous thrombosis	CVST as NB complication
Keyvanfar et al., 2025 [18]	Global review	Mixed	Synthesizes infarcts; MRA patterns	Vasculitis or endarteritis, septic emboli, CVT
Present case, 2026	Qatar	47 y, male	Right MCA territory infarct; M2 occlusion adjacent to right temporal lobe Brucella abscess	Direct mechanical compression of M2 segment by abscess, with likely contributory perilesional vasculopathy

MRV, magnetic resonance venography.

The proposed mechanism in this case warrants careful reflection. Cross-sectional imaging localized the M2 occlusion to the segment immediately abutting the abscess (Fig. 5), and MRA excluded large-vessel beading or other angiographic features of widespread vasculitis. Mechanical narrowing therefore offers the most parsimonious explanation for the territorial infarction. However, mass effect alone is unlikely to be the sole contributor. The perilesional inflammatory milieu surrounding a pyogenic Brucella abscess is rich in cytokines, granulomatous infiltrate, and endothelial injury mediators that can promote thrombogenesis within adjacent vessels [6, 8]. A localized inflammatory vasculopathy involving the M2 segment, occurring in parallel with extrinsic compression, is therefore biologically plausible and likely synergistic. Both mechanisms can converge to produce thrombotic occlusion, and the histopathological substrate would not be readily distinguishable on conventional angiographic imaging.

Brucella reaches the central nervous system through several established routes. Hematogenous dissemination during bacteremia is the most common, although direct extension from contiguous structures and lymphatic spread have also been described [4, 6]. Once intraparenchymal, intracellular survival within macrophages drives chronic granulomatous inflammation. The resultant lesion may evolve into a localized abscess when host containment fails, particularly in regions with rich vascular supply such as the perisylvian cortex. Adjacent arteries are exposed not only to extrinsic compression but also to inflammatory mediators that compromise endothelial integrity, creating conditions favorable for in situ thrombosis [6, 8, 14].

Diagnostic recognition was substantially delayed in this case. The patient lacked classical systemic features of brucellosis, and the language barrier deferred capture of the occupational exposure history. Inflammatory markers were only mildly elevated, and initial CSF findings were nonspecific. The diagnosis hinged on focused serological testing once livestock exposure was clarified. This sequence underscores a recurring lesson: in endemic settings, structured occupational and dietary history-taking should be embedded into routine stroke workup, and Brucella serology should be obtained early when intracranial infectious features accompany an ischemic event [3, 17].

Therapeutic decision-making in stroke complicated by intracranial abscess is nuanced. Standard secondary prevention with antiplatelet therapy and statin was administered alongside antimicrobial therapy. Anticoagulation was avoided because of the abscess and concern for hemorrhagic transformation. Surgical drainage was deferred in favor of medical management, supported by the lesion size, anatomic location, and absence of significant midline shift. Triple antimicrobial therapy with ceftriaxone, doxycycline, and rifampicin remains the most widely endorsed regimen for NB and yielded a favorable response in this patient [1, 4]. Optimal duration is not established, although most reports support continued oral therapy for 6–12 months guided by clinical and radiological response.

Several limitations warrant consideration. First, definitive microbiological confirmation of Brucella species within the abscess was not obtained, as surgical drainage was deferred; the diagnosis rested on positive serology and the compatible clinical and radiological picture. Second, distinguishing between purely mechanical compression and a coexisting vasculitic process is difficult without histopathological sampling, and the relative contribution of each remains uncertain. Third, advanced vessel-wall imaging that could have characterized the M2 segment more precisely was not available at presentation. Finally, follow-up was confined to short-term clinical and radiological assessment, and longer term outcomes are not yet available.

The findings carry several practical implications. Brucellosis should remain part of the diagnostic differential in patients with acute ischemic stroke and concurrent rim-enhancing intracranial lesions, particularly in endemic regions or with relevant occupational exposure. Vessel-wall MRI and high-resolution MRA may help characterize coexisting inflammatory vasculopathy in similar presentations. Future work should focus on prospective registries of NB-related stroke to better delineate the relative frequency of compressive, vasculitic, and embolic mechanisms, and on clarifying whether earlier surgical drainage influences cerebrovascular outcomes when an abscess abuts a major intracranial artery.

## Conclusion

This case demonstrates that NB can produce ischemic stroke through direct compression of a major intracranial artery by an adjacent abscess, expanding the recognized cerebrovascular spectrum of the disease. In endemic regions, brucellosis warrants inclusion in the differential diagnosis of unexplained cerebrovascular events, particularly when imaging reveals concurrent rim-enhancing lesions. Early recognition, focused serological testing, and prompt initiation of triple antimicrobial therapy are critical to limit irreversible neurological injury and improve outcomes.

## Statement of Ethics

This work was conducted in accordance with the Declaration of Helsinki (1964). This case report was approved by the Institutional Review Board at Hamad Medical Corporation, Doha, Qatar (MRC-04-25-1122). Written informed consent for publication of this case report and any accompanying images was obtained from the patient.

## Conflict of Interest Statement

The authors have no conflict of interest to declare.

## Funding Sources

The authors have not declared a specific grant for this research from any funding agency in the public, commercial or not-for-profit sectors. Open Access fee was provided by Qatar National Library. The funder had no role in the design, data collection, data analysis, and reporting of this study.

## Author Contributions

Ruba Adel Aweer: conceptualization, data curation, writing – original draft, and writing – review and editing. Majd A. AbuAlrob: supervision and writing – review and editing. Khaled Zammar and Ahmad Elsotouhy: supervision and data curation. All authors read and approved the final manuscript.

## Data Availability

To protect patient confidentiality, the data are not publicly available but can be obtained from the corresponding author upon request.
